# Validation of H5 influenza virus subtyping RT-qPCR assay and low prevalence of H5 detection in 2024–2025 influenza virus season

**DOI:** 10.1128/jcm.00415-25

**Published:** 2025-10-21

**Authors:** David J. Bacsik, Margaret G. Mills, Luke D. Monroe, Cassey Spring, Ailyn C. Perez-Osorio, Jonathan C. Reed, Ferric C. Fang, Lori Bourassa, Pavitra Roychoudhury, Katharine H. D. Crawford, Kevin Snekvik, Alexander L. Greninger

**Affiliations:** 1 Department of Laboratory Medicine and Pathology, University of Washington 7284, Seattle, Washington, USA; 2 Vaccine and Infectious Disease Division, Fred Hutchinson Cancer Center 7286, Seattle, Washington, USA; 3 Washington Animal Disease Diagnostic Laboratory, College of Veterinary Medicine, Washington State University 6760, Pullman, Washington, USA; 4 Department of Veterinary Microbiology and Pathology, College of Veterinary Medicine, Washington State University 6760, Pullman, Washington, USA; University of California, Davis, Davis, California, USA

**Keywords:** H5N1, influenza, PCR, NAAT, surveillance, subtyping

## Abstract

A sustained outbreak of H5N1 influenza virus among wild fowl and domestic livestock has caused more than 70 zoonotic infections in humans in North America, including two deaths. The United States Centers for Disease Control and Prevention has recommended rapid H5 subtyping for all hospitalized cases with influenza A virus infection to enable prompt initiation of antiviral treatment, as well as infection prevention and implementation of public health measures to control spread. To address these needs, we developed a qualitative multiplex RT-qPCR assay to subtype H5 influenza virus in nasal, nasopharyngeal, and conjunctival specimens with a limit of detection of 250 copies/mL. No cross-reactivity was observed with other common respiratory viruses, including seasonal H3N2 and H1N1 influenza A viruses. We retrospectively subtyped 590 influenza A virus-positive clinical specimens with Ct values less than 31 processed by University of Washington labs between March 2024 and February 2025, including 512 specimens collected during the 2024–2025 influenza season, and detected no H5 positives. After clinical implementation, we performed 150 clinically ordered H5 subtyping tests between February and April 2025 and again detected no positives. This work enhances clinical pandemic preparedness activities and highlights the exceedingly low prevalence of H5N1 influenza virus during the 2024–2025 respiratory season.

**IMPORTANCE:**

The spread of H5N1 influenza virus in the United States has led to the culling of almost 200 million birds, infected cow herds across 17 states, and resulted in 70 human infections as of July 2025. Rapid PCR subtyping of H5 influenza virus is critical to inform hospital infection prevention and public health to enable containment of viral transmission. Here, we report the design, validation, and clinical implementation of a qualitative multiplex H5-subtyping RT-qPCR assay for nasopharyngeal, nasal, and conjunctival swab specimens. Additionally, we offer the largest reported study of H5 subtyping of influenza A virus-positive specimens in the United States to date. No H5 infections were detected in 740 samples collected between March 2024 and April 2025 from patients with confirmed influenza A virus infection in a large academic medical system in Seattle, WA.

## INTRODUCTION

H5N1 influenza virus has circulated continuously among birds in North America since 2021 (1). Since late 2023, a sustained outbreak of clade 2.3.4.4b H5N1 influenza A virus has spread among dairy and poultry farms in the United States (2). Currently, two genotypes within clade 2.3.4.4b co-circulate simultaneously: genotype B3.13, which has demonstrated sustained transmission among cattle (2, 3), and genotype D1.1, which circulates in wild bird populations and has caused repeated introductions into livestock, including domestic poultry (4) and dairy cattle (5).

Human infections of both genotypes have been detected, with the first outbreak-related case identified in April 2024 (6). To date, more than 70 cases have been reported in North America (7). Although most cases have not required hospitalization, three cases of critical illness have been reported, including deaths in Louisiana and Durango, Mexico (8–10). Conjunctivitis has been the most common symptom reported (11). Exposure to infected livestock is a significant risk factor, and farm workers have been disproportionately impacted (11). In a serological survey of dairy workers on H5N1-infected farms, 7% had evidence of recent infection with a subtype H5 influenza virus (12), suggesting that the prevalence of infection in this population could be much higher than has been recognized.

Prompt identification of H5N1 influenza virus infection is necessary to provide appropriate medical care and to contain potential further spread of the virus. The Centers for Disease Control and Prevention (CDC) recommends that all hospitalized patients with influenza A virus infections have subtyping performed within 24 h of admission—especially patients who are critically ill (13). Because H5N1 influenza virus can cause severe illness, and because circulating strains remain sensitive to antiviral medications, treatment with oseltamivir is indicated for symptomatic patients with suspected H5N1 influenza virus infections (14). Furthermore, airborne precautions and patient isolation are recommended for infection control (15).

In addition to informing clinical management, molecular diagnosis of H5N1 improves influenza surveillance. Though efforts have been made to increase surveillance of non-subtypeable influenza, much of the current surveillance infrastructure has focused on monitoring epidemiologically linked cases with known exposure to infected animals, and it is possible that cases without recognized risk factors have gone undetected (16). This pattern has occurred during previous outbreaks, including SARS-CoV-2 (17). To provide unbiased monitoring of H5N1 infections among all influenza cases, integration within health systems is required. To increase testing capacity, the CDC has issued a call (18) requesting the development and implementation of laboratory-developed tests to detect subtype H5 influenza virus (19–21).

Here, we report our validation of a qualitative multiplex RT-qPCR assay for H5 influenza virus subtyping in nasopharyngeal, nasal, and conjunctival swabs. We have used the assay to test 740 influenza A virus-positive specimens processed by the University of Washington Laboratories between March 2024 and April 2025, detecting no cases of H5 influenza virus to date.

## MATERIALS AND METHODS

### Clinical specimens and associated metadata

Residual deidentified nasopharyngeal swabs, nasal swabs, and conjunctival swabs from the UW Virology Laboratory were used for validation. Retrospective subtyping was performed by testing available residual influenza A virus-positive respiratory specimens from UW Medicine with collection dates of March 2024 or later. These dates were chosen based on the date of the first human H5N1 infection reported in the United States (22). Available samples had been previously collected for genomic surveillance and had Ct values less than 31. After assay validation and implementation at UW Medicine, test results and metadata were obtained from the laboratory information system. This study was approved by the UW Institutional Review Board with a consent waiver (STUDY00010205).

### H5 RNA templates

Because clinical specimens containing H5N1 influenza virus were not readily available during initial test validation, we employed three alternative sources of H5 RNA: two sets of synthetic RNA templates and a collection of H5-positive RNA samples extracted from animal specimens. The first set of synthetic RNAs was transcribed *in vitro*, referred to as “IVT RNA templates.” The H5 RNA was transcribed from a gBlock containing the *HA* coding sequence of the clade 2.3.4.4b H5N1 virus strain A/white-tailed eagle/Hokkaido/20220322001/2022 (File S1) using the New England Biosystems HiScribe T7 RNA Synthesis Kit (E2040). The M RNA was prepared as previously described (23). A second set of synthetic RNA was obtained from the National Institute of Standards and Technology (NIST) as H5N1 (Avian Influenza) Synthetic RNA Fragments (Research-Grade Test Material 10263). The NIST RNA set includes synthetic RNAs encoding the *HA*, *M*, and *NA* genes of the clade 2.3.4.4b H5N1 virus strain A/American Wigeon/South Carolina/22/2021 in a background of 5 ng/µL Jurkat cell RNA.

Authentic H5N1-positive RNA samples (*n* = 7) were obtained from the Washington State University Animal Disease Diagnostic Laboratory. The samples were previously extracted from a variety of animal-source specimens, including mammalian respiratory swabs, mammalian tissue, bulk milk, and avian oropharyngeal/cloacal swabs (Table S1). All were known to be influenza A virus-positive by prior RT-qPCR and known to contain H5N1 RNA by prior whole-genome sequencing.

### Inactivated H5N1 viruses

Additional validation was performed using inactivated H5N1 viruses representing the B3.13 and D1.1 genotypes. For genotype B3.13, inactivated H5N1 reference material was obtained from BEI Resources (NR-59886), containing tissue-culture adapted strain A/bovine/Ohio/B24OSU-439/2024 and cell lysate that had been inactivated by gamma-irradiation.

For genotype D1.1, H5N1 influenza virus isolate A/Washington/239/2024 was obtained from the CDC, passaged, and inactivated. Before passaging, 3.5 × 10^6^ MDCK cells were seeded in a T75 flask in growth media consisting of MEM with Earle’s salts and 2 mM glutaMAX (Thermo 42360) supplemented with 10% vol/vol heat-inactivated FBS (Thermo A3840002), 1% vol/vol Pen-Strep (Thermo 151401), and 1% vol/vol NEAA (Thermo 111400) and incubated overnight. The next day, virus inoculum was prepared by diluting the virus stock in infection media containing MEM with Earle’s salts and 2 mM glutaMAX (Thermo 42360), 0.3% wt/vol BSA (Sigma-Aldrich A8412), 1% vol/vol Pen-Strep (Thermo 151401), 1% vol/vol NEAA (Thermo 111400), and 0.4 µg/mL TPCK-treated trypsin (Thermo NC9783694). The cells were washed with plain MEM to remove FBS and infected at an MOI of 0.01 based on the titer provided with the stock (2.0 × 10^8^ TCID50/mL) in 2 mL of infection media. The virus was allowed to adsorb for 1 h, redistributing the inoculum every 20 min. After adsorption, 8 mL of infection media was added for a final volume of 10 mL. At 2 days post-infection, with more than 90% of the cell monolayer exhibiting cytopathic effect, the supernatant was collected, separated from cellular debris by centrifugation at 500 × *g* for 10 min, and aliquoted. Virus stocks were titered by TCID50 assay on MDCK cells using eight replicates per dilution, and cytopathic effect was scored at 5 days post-infection. TCID50/mL estimates were determined using the Spearman-Karber method (24).

The virus was inactivated using a validated procedure approved by the University of Washington Institutional Biosafety Committee. Briefly, viral supernatants were heat-treated in 400 µL aliquots at 65°C for 30 minutes. For each aliquot, inactivation was confirmed by testing 100 µL of the inactivated material by TCID50 assay. Positive controls (not heat-treated) and negative controls (no virus) were included with each assay.

### RNA extraction

RNA was extracted from clinical specimens on a Roche MagNA Pure 96 instrument using the Viral NA Small Volume Kit (06543588001). For each sample, 200 µL of specimen was used as input and RNA was eluted in 50 µL of elution buffer. An exogenous RNA template was included with the lysis buffer as an internal control (Table 1).

**TABLE 1:** Primer and probe sequences and concentrations^*a*^

Target	Oligo name	Sequence	Final concentration
**H5-1**	H5 Forward PrImer 1	TGGAAAGTGTGAGAAATGGGACGT	500 nM
	H5 Reverse Primer 1	TGCTAGGGAACTTGCCGCTG	500 nM
	H5 Probe 1 (FAM)	FAM-TGACTACCCGCAGTATTCAGAAGAAGCAAGACTAA-ZEN/IABK	500 nM
**H5-2**	H5 Forward Primer 2	TGGGTACCATCATAGCAATGAGCA	500 nM
	H5 Reverse Primer 2	AACTCCCTTCCAACTGCCTCAAA	500 nM
	H5 Probe 2 (FAM)	FAM-TGGGTACGCTGCGGACAAAGAATCCA-ZEN/IABK	500 nM
**M**	M Forward Primer 1	CAAGACCAATCYTGTCACCTCTGAC	250 nM
	M Forward Primer 2	CAAGACCAATYCTGTCACCTYTGAC	250 nM
	M Reverse Primer 1	GCATTYTGGACAAAVCGTCTACG	250 nM
	M Reverse Primer 2	GCATTTTGGATAAAGCGTCTACG	250 nM
	M Probe (SUN)	SUN-TGCAGTCCTCGCTCACTGGGCACG-ZEN/IABK	500 nM
**Internal control**	EXO Forward Primer	AATTGGAAGTGGCGGAAGAA	100 nM
	EXO Reverse Primer	GGAACCTAAGACAAGTGTGTTTATGG	200 nM
	EXO Probe (Cy5)	Cy5-AGCTATTGCAAACGCCATCGCACAA-BHQ	500 nM

^*a*^ Targets “H5-1” and “H5-2” are located on the H5 influenza *HA* gene. Target “M” is located on the influenza A *M* gene. Target “Internal Control” is located on an exogenous RNA transcript which is added during extraction and encodes a *TMP1* gene.

### Reverse transcription quantitative PCR reaction conditions

Multiplex reverse transcription quantitative PCR (RT-qPCR) was performed using the AgPath-ID One-Step RT-PCR Kit (AM1005). For each reaction, 5 µL of extracted RNA was added to a reaction mix containing 12.5 µL AgPath Master Mix, 1 µL AgPath Enzyme, and primers and probes at specified concentrations (see Table 1); the total reaction volume was 25 µL. Reactions were run on ABI 7500 Thermocyclers. Cycle parameters were 45°C for 10 min, 95°C for 10 min, and 45 cycles of 95°C for 15 s, followed by 60°C for 45 s. ROX normalization and automatic baseline were used for all fluorophores. The threshold values were 0.18 for the H5 target (FAM), 0.16 for the influenza A target (VIC), and 0.1 for the internal control target (Cy5). Ct values less than 45 were considered positive.

### Reverse transcription droplet digital PCR reaction conditions

Reverse transcription droplet digital PCR (RT-ddPCR) was performed with the BioRad One-Step RT-ddPCR Advanced Kit for Probes (1864022) using the H5-2 and M primers and probes from the RT-qPCR assay (Table 1). Each 25 µL reaction was prepared by combining 5 µL of extracted RNA with 20 µL of master mix containing 6.25 µL Super Mix, 2.5 µL RT enzyme, 1.25 µL DTT, primers at a final concentration of 900 nM for H5-2 and 450 nM each for M, and probes at a final concentration of 250 nM. 20 µL of the total reaction mix was used for droplet generation using a BioRad Automated Droplet Generator, and 40 µL of droplet suspension was transferred to a 96-well plate. Reactions were run on a BioRad C1000 Touch thermocycler, using the following conditions: 50°C for 60 min, 95°C for 10 min, 40 cycles of 95°C for 30 s, followed by 60°C for 1 min, and 98°C for 10 min. Fluorescence was measured on a BioRad QX600 Digital Droplet Reader using the absolute quantification method. Samples with more than 50% positive droplets fell outside of the quantitative range and were excluded from analysis.

The absolute concentration of the IVT RNA and NIST RNA templates was measured by RT-ddPCR as described above. The absolute copy number of the unitless BEI H5N1 reference material and the inactivated H5N1 virus was measured following RNA extraction as described above.

### Computational analysis of primer and probe sequences

To assess the similarity between the chosen oligonucleotides and contemporary North American viruses, computational analysis was performed. All H5 HA sequences collected in North America between 1 January 2024 and 31 January 2025 were downloaded from NCBI (25). The sequences were aligned using MAFFT (26). Binding regions were annotated, and each primer or probe was analyzed independently. Sequences that lacked coverage at one or more nucleotides in the binding site were excluded. The number of mismatches was counted; ambiguous nucleotides were conservatively treated as mismatches.

### Limit of detection

The limit of detection (LOD) of the complete assay, including extraction, was assessed using inactivated H5N1 virus (A/Washington/239/2024) spiked into pooled, influenza virus-negative nasopharyngeal swab specimens. The initial LOD was estimated using a 10-fold dilution series ranging from 1:10^2^ to 1:10^8^, with RNA extracted from four replicates per concentration. Additional replicates were prepared for comparison with the complete CDC assay protocol (see “Comparison to CDC Influenza A/H5 Subtyping Kit” below). The LOD was confirmed using a 4-fold serial dilution around the initial limit, with RNA extracted from 20 replicates of each concentration. The 95% LOD was reported as the lowest concentration with at least 19 of the replicates yielding a positive result. The absolute LOD of the multiplex RT-qPCR reaction was determined using IVT H5 (stock 1.07 × 10^6^ copies/µL by RT-ddPCR using H5-2 primers and probes) and NIST M (1.47 × 10^5^ copies/µL by RT-ddPCR using M primers and probes) RNA templates. Ten microliters of each RNA template was combined and diluted in 80 µL of AE buffer (10 mM Tris-HCl and 0.5 mM EDTA at pH 9.0; Qiagen #19077). This stock was diluted 10-fold in AE buffer for initial LOD determination and 2-fold around the initial LOD for confirmation. Twenty replicates of each concentration were tested, and the 95% LOD was reported as the lowest concentration with at least 19 of the replicates yielding a positive result.

### Assay sensitivity and matrix compatibility

Assay sensitivity was assessed using H5N1-positive RNA samples extracted from animal specimens (*n* = 7) and contrived H5N1-positive specimens spiked with inactivated B3.13 virus (*n* = 69) or D1.1 virus (*n* = 160). To prepare low-positive specimens containing genotype B3.13 virus, inactivated A/bovine/Ohio/B24OSU-439/2024 virus, which was received unitless from BEI, was added at a ratio of 1:50,000 by volume to the nasal and conjunctival specimens, and 1:10,000 to the nasopharyngeal specimens. Specimens with at least 220 µL volume were tested individually, while specimens with less volume were pooled and aliquoted. In total, 29 nasal specimens were prepared (20 individual and 9 pooled); 20 conjunctival specimens were prepared (7 individual and 13 pooled); and 20 nasopharyngeal specimens were prepared (20 individual and 0 pooled).

To prepare H5N1-positive specimens containing genotype D1.1 virus, inactivated A/Washington/239/2024 virus was added to pooled nasopharyngeal, nasal, or conjunctival specimens or to AE buffer. Three levels of H5N1-positive specimens were prepared with each matrix, targeting low (400 TCID50/mL), medium (20,000 TCID50/mL), and high (1,000,000 TCID50/mL) titers of H5N1 virus. Twenty replicates were prepared of the low-concentration specimens, and 10 replicates were prepared of the medium- and high-concentration specimens. Confidence intervals (95% CIs) for measures of agreement were calculated using the Clopper-Pearson exact method.

### Assay specificity

Three types of H5-negative specimens were used to assess specificity: nasopharyngeal swabs (*n* = 20), nasal swabs (*n* = 29), and conjunctival swabs (*n* = 18). The respiratory specimens were confirmed as negative for influenza A/B virus by Cepheid Xpert Xpress CoV-2/Flu/RSV plus or Hologic Panther Fusion Flu A/B/RSV assays. Conjunctival swabs were collected for HSV and/or VZV testing. Specificity was further evaluated using specimens known to contain common respiratory viruses by clinical testing via Cepheid or Hologic respiratory panel tests or whole-genome sequencing. These included influenza A virus subtypes H3N2 (*n* = 21) and H1N1 (*n* = 17), influenza B virus (*n* = 3), respiratory syncytial virus (*n* = 7), and SARS-CoV-2 (*n* = 10).

### Comparison to CDC influenza A/H5 subtyping kit

Since only one RT-qPCR assay is FDA-cleared for H5N1 testing, we compared our multiplex RT-qPCR assay with the CDC’s Influenza A/H5 Subtyping Kit (Version 4, #FluIVD03-11). To assess the relative performance of the complete assays, including extraction, replicate samples were prepared containing inactivated A/Washington/239/2024 virus spiked into pooled nasopharyngeal specimens (see “Limit of Detection” above). RNA was extracted following the CDC H5 Subtyping Kit protocol, which differs from the extraction protocol used in our multiplex assay. Using a Roche MagNA Pure 96 instrument with the Viral NA Small Volume Kit (06543588001), 100 µL of specimen was combined with 350 µL of External Lysis Buffer (06374913001) and the entire volume (450 µL) was used as input. RNA was eluted in 100 µL of elution buffer. The CDC RT-qPCR reactions were run according to the manufacturer’s protocol using the Invitrogen SuperScript III Platinum One-Step Quantitative RT-PCR System on the ABI 7500 FAST thermocycler. For each singleplex reaction in the CDC kit, 5 µL of nucleic acid template was added to a 20 µL reaction mix containing 12.5 µL of 2× PCR master mix, 5.5 µL water, 0.5 µL enzyme, and 1.5 µL combined primer/probe mix prepared according to the kit protocol; total volume was 25 µL.

To compare the performance of the RT-qPCR reactions on isolated RNA, the CDC H5 Subtyping reactions and the novel multiplex reactions were run in parallel on the same set of isolated RNA templates. For the IVT RNA and NIST RNA templates, 10-fold dilution series were prepared by combining H5 and M synthetic RNA at a ratio of 1:1 by volume and serially diluting the template mix in AE buffer. Inactivated A/bovine/Ohio/B24OSU-439/2024 virus was initially diluted 1:100 in PBS, and a 10-fold dilution series in PBS was prepared from this stock with RNA extracted as described above. The absolute copy number of each template stock was determined by RT-ddPCR after dilution (and extraction, in the case of the inactivated virus). RT-qPCR reactions were run as described for each assay.

## RESULTS

### Design and computational analysis of primer and probe sequences

A three-channel multiplex RT-qPCR reaction was designed for qualitative detection of subtype H5 influenza virus. The primary target was the H5 influenza virus *HA* gene. To reduce the risk of false-negative results due to viral evolution (27) or reagent failure (28), primers and probes were selected to target two non-overlapping regions of the gene. One H5 target (H5-1) generated a 149 bp product from positions 1,481 to 1,629 of the HA CDS (GenBank accession no. LC730539; Fig. S1) using oligonucleotides adapted from designs by Shu et al. (29). The other H5 target (H5-2) used oligonucleotides designed by Sahoo et al. (19) and adapted from sequences originally designed by the Hong Kong Centre for Health Protection (30). This target generated a 144 bp product from positions 1,101 to 1,244 of the HA CDS. As a pan-influenza A virus control, a highly conserved region of the influenza A *M* gene was targeted using primers and probes designed by the CDC National Center for Immunization and Respiratory Diseases (31). Oligonucleotides targeting an exogenous RNA template encoding the *TMP1* gene from the marine species *Podocornye carnea* were included as an internal control (32).

Because materials for validation were highly limited when assay development began, the primers and probes were designed to bind to a reference material containing inactivated H5N1 virus from 2009 (BEI Resources NR-59421) with a divergent *HA* sequence. As a result, genotype B3.13 viruses carry two mismatches in H5 Probe 1 and no mismatches in H5 Probe 2; and genotype D1.1 viruses carry three mismatches in H5 Probe 1 and one mismatch in H5 Probe 2 (Fig. S1). Despite these sequence differences, the probes remain functional with contemporary templates. The same mismatches are present in the IVT RNA target, which is reliably detected by either probe alone (Table S2).

The similarity between the oligonucleotide sequences and contemporary North American H5 influenza virus sequences was assessed computationally. A total of 2,788 H5 *HA* sequences collected in North America between 1 January 2024, and 31 January 2025, were retrieved from NCBI GenBank (Table 2). Of the six oligonucleotides targeting H5 *HA*, five matched recent sequences closely, with two or fewer mismatches in 99% of sequences. One oligonucleotide (H5 Probe 1) displayed two mismatches in 92% of recent H5 sequences, and three or more mismatches in 8% of sequences—predominantly genotype D1.1 strains.

**TABLE 2:** Similarity of oligonucleotide sequences and recent H5 HA sequences collected in North America^*a*^

Name	Sequences	0 Mismatch	1 Mismatch	2 Mismatch	3+ Mismatch	0 Mismatch %	≤2 Mismatch %	3+ Mismatch %
All sequences	2,788							
H5 Forward 1 Primer	2,759	2,732	27	0	0	99.0%	100.0%	0.0%
H5 Forward 2 Primer	2,782	2,736	38	1	7	98.3%	99.7%	0.3%
H5 Probe 1	2,759	0	3	2,537	219	0.0%	92.1%	7.9%
H5 Probe 2	2,781	2,615	154	0	12	94.0%	99.6%	0.4%
H5 Reverse 1 Primer	2,755	2,589	154	7	5	94.0%	99.8%	0.2%
H5 Reverse 2 Primer	2,780	2709	55	4	12	97.4%	99.6%	0.4%

^*a*^ Primer and probe sequences used in the multiplex RT-qPCR assay were computationally compared to recently circulating H5 *HA* sequences available on NCBI.

### Estimates of template copy number by Ct value

To determine the relationship between the Ct value produced by the RT-qPCR reaction and the absolute copy number of template molecules, a dilution series of RNA extracted from inactivated H5N1 virus A/Washington/239/2024 was tested by RT-qPCR and RT-ddPCR in parallel (Fig. S2). Dilutions between 1:10^4^ and 1:10^6^ yielded RT-ddPCR results in the quantitative range, providing a range of three orders of magnitude for comparison (Table S3). A strong linear relationship was observed for both the H5 target (*R*^2^ = 0.99) and the M target (*R*^2^ = 0.98). While the clinical assay is designed to be qualitative and not quantitative, the absolute copy number of templates in the multiplex RT-qPCR reaction can be estimated from the Ct value using a linear model fit to these data (Table S4).

### Limit of Detection

The absolute LOD of the RT-qPCR reaction was measured using a 2-fold serial dilution of H5 and M IVT RNA templates in AE buffer (Table 3). The 95% LOD (LOD95) was measured by identifying the lowest concentration with at least 19 out of 20 replicates detected (Table S5). The LOD95 for the H5 target was 4.7 copies per reaction and 36.3 copies per reaction for the M target.

**TABLE 3:** Absolute limit of detection of multiplex RT-qPCR reaction^*a*^

H5 target	Targeted copies/rxn	4.7	2.4	1.2	0.6
	Avg. Ct	34.6	37.4	38.2	39.0
	Std. Dev.	1.1	2.2	1.9	0.9
	% CV	3.3%	5.8%	5.0%	2.3%
	# Detected	20	18	15	8
IAV M target	Targeted copies/rxn	72.5	36.3	18.1	9.1
	Avg. Ct	35.4	36.3	38.7	40.2
	Std. Dev.	0.8	1.1	1.2	1.8
	% CV	2.2%	3.1%	3.0%	4.4%
	# Detected	20	19	17	10

^*a*^ *N*=20. Targeted copies per reaction are based on the absolute concentration of stock measured by RT-ddPCR. Summary statistics were calculated using detected samples; non-detected samples were excluded.

**Fig 1:**
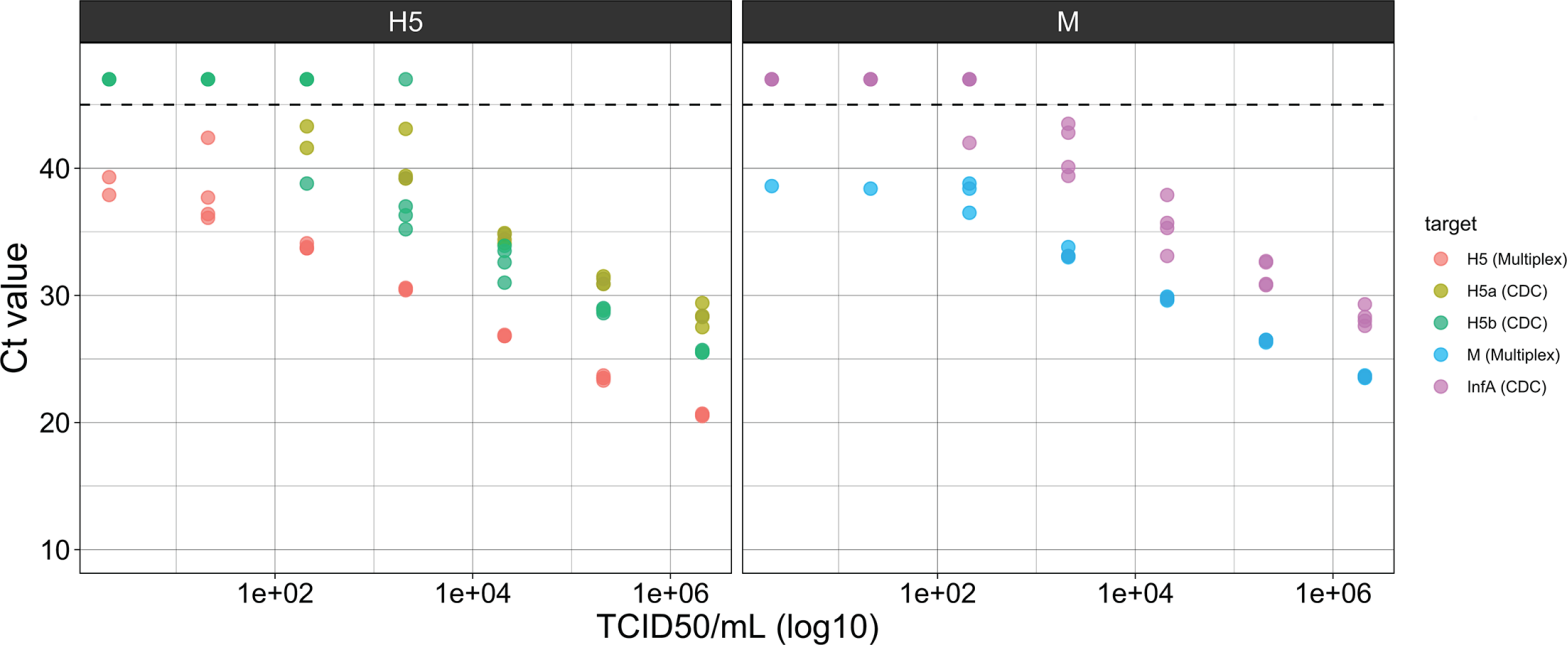
Comparison of multiplex RT-qPCR assay and CDC Influenza A/H5 Subtyping assay. *N* = 4. Ten-fold serial dilutions of inactivated H5N1 virus A/Washington/239/2024 in nasopharyngeal swab matrix were processed following each assay’s specified extraction and RT-qPCR protocol. Points appearing above the dashed line were not detected after 45 cycles of PCR.

The LOD of the complete assay including extraction was measured using inactivated H5N1 virus (A/Washington/239/2024) diluted in pooled nasopharyngeal swab specimens, with independent replicates starting at extraction (Table 4). The LOD95 for the H5 target was 24 TCID50/mL and the LOD95 for the M target was 390 TCID50/mL (Tables S6 and S7). Based on the Ct values observed, we estimate an absolute LOD95 in nasopharyngeal specimens of 5.0 copies per reaction for the H5 target (250 copies/mL) and 12.3 copies per reaction for the M target (615 copies/mL), which agree closely with the absolute limits measured using synthetic RNA templates in buffer. The assay displayed linear performance across 5 logs of input with *R*^2^ values of 0.998 for the H5 target and 0.996 for the M target (Fig. 1). Efficiency was 99.0% for the H5 target and 104.5% for the M target.

**TABLE 4:** Limit of detection of clinical assay including extraction^*a*^

	TCID50/mL	390	98	24	6
H5 target	Estimated copies/rxn	89.5	23.3	5.0	2.0
	# Detected	20	20	20	14
	Avg. Ct	33.9	35.9	38.3	39.2
	Std. Dev.	0.24	0.60	0.90	1.02
	% CV	0.72	1.68	2.35	2.61
IAV M target	Estimated copies/rxn	12.3	2.5	0.7	0.2
	# Detected	20	11	3	1
	Avg. Ct	36.7	37.1	37.8	38.2
	Std. Dev.	0.85	0.68	0.58	N/A^*b*^
	% CV	2.32	1.83	1.54	N/A^*b*^

^*a*^ *N*=20. Estimated copies per reaction were calculated using the previously determined linear relationship between Ct value and copy number. Summary statistics were calculated using detected samples; non-detected samples were excluded.

^*b*^ N/A, not applicable.

### Sensitivity and matrix compatibility

Sensitivity was assessed across a range of virus concentrations, matrices, and genotypes using clinical specimens spiked with inactivated H5N1 virus. For the B3.13 genotype, we generated low-concentration H5-positive samples by adding inactivated A/bovine/Ohio/B24OSU-439/2024 virus to influenza virus-negative nasal and conjunctival swab specimens at a ratio of 1:50,000 by volume and to nasopharyngeal specimens at a ratio of 1:10,000 (Table 5). Following extraction and RT-qPCR testing, mean H5 Ct values were 34.7 for nasopharyngeal specimens (estimated 50.5 copies per reaction), 36.7 for nasal specimens (estimated 16.7 copies per reaction), and 35.9 for conjunctival specimens (estimated 22.0 copies per reaction). All 20 nasopharyngeal swab specimens and all 29 nasal swab specimens tested positive for the H5 target, demonstrating 100% positive agreement (Table S8). Of the conjunctival swabs, 19 out of 20 samples tested positive for the H5 target, demonstrating 95% positive agreement.

**TABLE 5:** Assay sensitivity with B3.13 genotype^*a*^

Specimen type	Total (*N*)	H5 positive (N)	IAV M positive (N)	Agreement (95% CI)	Mean Ct values (SD)
Nasopharyngeal	20	20	20	H5: 100% (83.2–100) M: 100% (83.2–100)	H5: 34.7 (0.6) M: 34.6 (1.1)
Nasal	29	29	25	H5: 100% (88.3–100) M: 86.2% (68.9–95.6)	H5: 36.3 (1.06) M: 37.3 (1.0)
Conjunctival	20	19	18	H5: 95% (75.1–99.9) M: 90% (68.7–98.1)	H5: 35.9 (0.7) M: 37.2 (1.0)

^*a*^ Sensitivity was assessed using contrived low-positive clinical specimens spiked with inactivated genotype B3.13 virus (A/bovine/Ohio/B24OSU-439/2024). Summary statistics were calculated using detected samples; non-detected samples were excluded.

For the M target, all 20 of the nasopharyngeal swab specimens tested positive with a mean Ct value of 34.6 (estimated 48.7 copies/reaction), demonstrating 100% positive agreement. Twenty five of the 29 nasal swab specimens tested amplified the M target with a mean Ct value of 37.3 (estimated 6.7 copies per reaction), demonstrating 86% positive agreement. Eighteen of the 20 conjunctival swab specimens amplified the M target with a mean Ct value of 37.2 (estimated 7.2 copies per reaction), demonstrating 90% positive agreement.

**Fig 2:**
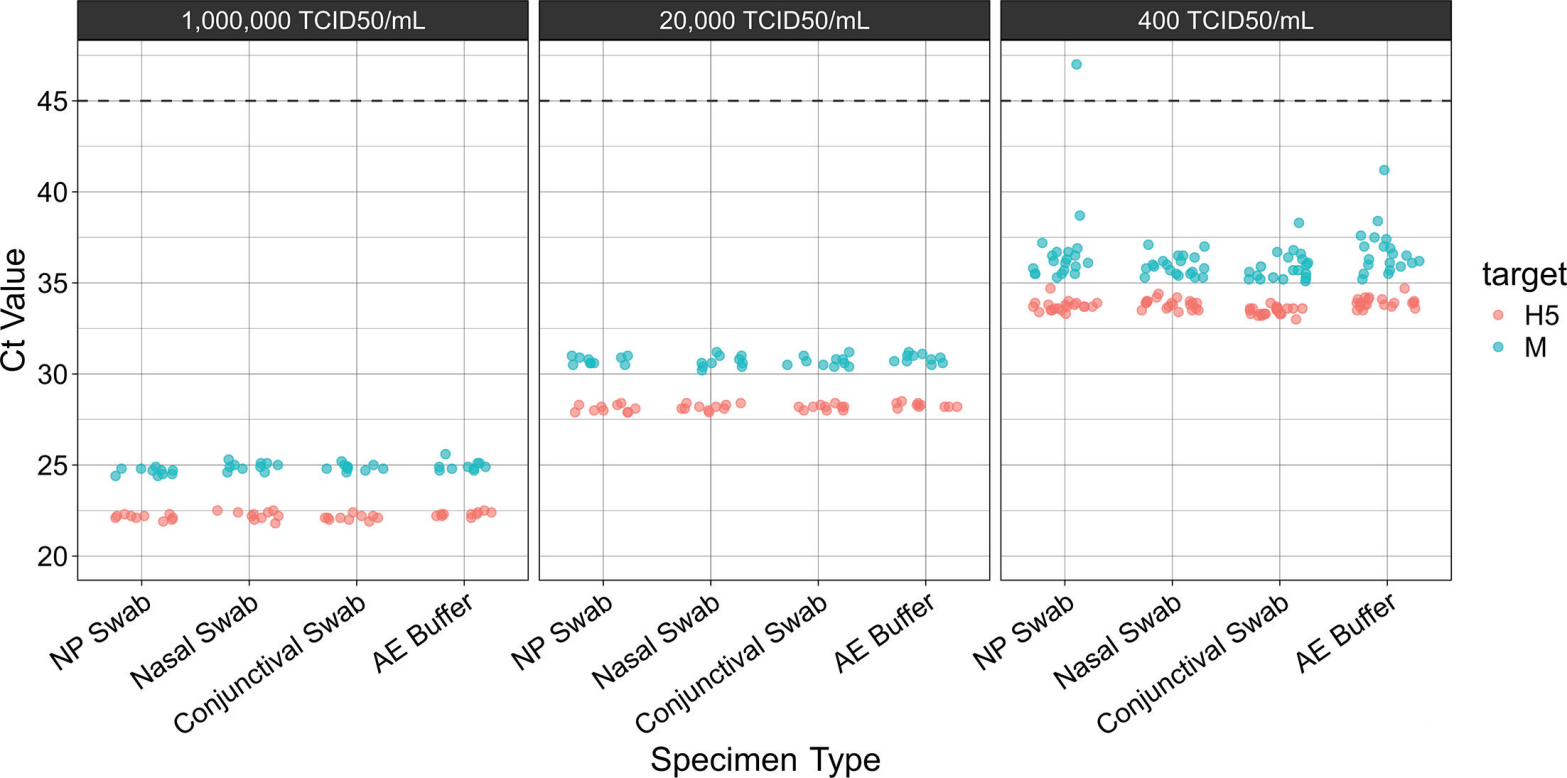
Effect of matrix on Ct values in low-, medium-, and high-concentration contrived specimens. Inactivated H5N1 virus A/Washington/239/2024 was spiked into pooled, influenza virus-negative clinical specimens or AE buffer, extracted, and tested by RT-qPCR. For low-titer specimens, *N* = 20. For medium- and high-titer specimens, *N* = 10. Points appearing above the dashed line were not detected after 45 cycles of PCR.

For the D1.1 genotype, all three matrices and AE buffer were spiked with inactivated H5N1 virus (A/Washington/239/2024) to create contrived positives at low, medium, and high titer (Table 6). The mean Ct values for the H5 target were 33.7 for the low-concentration specimens, 28.2 for the medium-concentration specimens, and 22.2 for the high-concentration specimens (estimated at 100, 4,500, and 284,000 copies per reaction, respectively). For the M target, the mean Ct values were 36.3, 30.7, and 24.8, respectively (estimated at 15, 850, and 64,000 copies per reaction, respectively). All specimens tested positive for the H5 target, demonstrating 100% positive agreement across all matrices and virus concentrations (Table S9). For the M target, one low-positive nasopharyngeal swab specimen falsely tested negative, demonstrating 95% positive agreement; all other matrices demonstrated 100% positive agreement. No strong matrix effects were observed. For all concentrations, Ct values were similar across specimen types, including AE buffer (Fig. 2).

**TABLE 6:** Assay sensitivity across a range of concentrations and matrices with D1.1 genotype^*a*^

Virus concentration	Specimen type	Total (*N*)	H5 positive (N)	IAV M positive (*N*)	Agreement (95% CI)	Mean Ct values (SD)
Low (400 TCID50/mL)	Nasopharyngeal	20	20	19	H5: 100% (83.2–100) M: 95% (75.0–99.9)	H5: 33.7 (0.3) M: 36.8 (0.8)
	Nasal	20	20	20	H5: 100% (83.2–100) M: 100% (83.2–100)	H5: 33.8 (0.3) M: 36.0 (0.5)
	Conjunctival	20	20	20	H5: 100% (83.2–100) M: 100% (83.2–100)	H5: 33.5 (0.2) M: 35.9 (0.8)
	AE buffer	20	20	20	H5: 100% (83.2–100) M: 100% (83.2–100)	H5: 33.9 (0.3) M: 36.7 (1.3)
Medium (20,000 TCID50/mL)	Nasopharyngeal	10	10	10	H5: 100% (69.2–100) M: 100% (69.2–100)	H5: 28.1 (0.2) M: 30.7 (0.2)
	Nasal	10	10	10	H5: 100% (69.2–100) M: 100% (69.2–100)	H5: 28.2 (0.2) M: 30.7 (0.3)
	Conjunctival	10	10	10	H5: 100% (69.2–100) M: 100% (69.2–100)	H5: 28.2 (0.1) M: 30.7 (0.3)
	AE buffer	10	10	10	H5: 100% (69.2–100) M: 100% (69.2–100)	H5: 28.3 (0.1) M: 30.9 (0.2)
High (1,000,000 TCID50/mL)	Nasopharyngeal	10	10	10	H5: 100% (69.2–100) M: 100% (69.2–100)	H5: 22.1 (0.1) M: 24.6 (0.2)
	Nasal	10	10	10	H5: 100% (69.2–100) M: 100% (69.2-100)	H5: 22.2 (0.2) M: 24.9 (0.2)
	Conjunctival	10	10	10	H5: 100% (69.2–100) M: 100% (69.2–100)	H5: 22.1 (0.1) M: 24.9 (0.2)
	AE buffer	10	10	10	H5: 100% (69.2–100) M: 100% (69.2–100)	H5: 22.3 (0.1) M: 25.0 (0.3)

^*a*^ Sensitivity was assessed using contrived clinical specimens spiked with inactivated genotype D1.1 virus (A/Washington/239/2024) at low, medium, or high titer. Summary statistics were calculated using detected samples; non-detected samples were excluded.

We obtained 7 animal RNA samples that were known to contain authentic H5N1 RNA by whole-genome sequencing and included both genotype B3.13 (*n* = 2) and genotype D1.1 (*n* = 1) genomes. All 7 samples amplified the H5 target with a mean Ct value of 24.4, demonstrating 100% positive agreement (Table S1). One B3.13 sample failed to amplify the pan-influenza A virus M target and had a late H5 Ct value of 38.2. Analysis of this sample’s sequence (BioSample SAMN41892145) revealed two mismatches in H5 Probe 1, one mismatch in M Reverse Primer 1, and two mismatches in M Reverse Primer 2. Given that the same mismatch profiles were present in many samples that successfully amplified both targets (e.g., BioSample SAMN46070075), the high Ct values are likely a result of sample degradation, rather than primer mismatch.

### Specificity

Three types of clinical specimens were evaluated for compatibility with the multiplex RT-qPCR assay (Table 7). Residual nasopharyngeal, nasal, and conjunctival swab specimens known to be influenza A virus-negative were extracted and tested. All specimens tested negative for both the H5 and M targets and tested positive for the internal control (Table S10).

**TABLE 7:** Specificity of multiplex RT-qPCR assay^*a*^

Sample type	Total (***N***)	H5 positive (***N***)	IAV M positive (***N***)	Internal control positive (***N***)	H5 Ct value (mean)	IAV M Ct value (mean)	Internal control Ct value (mean)	Agreement (95% CI)
**Influenza virus-negative clinical specimens**								
Nasopharyngeal swab	20	0	0	20	NDET	NDET	29.3	100% (83.2–100)
Nasal swab	29	0	0	29	NDET	NDET	29.4	100% (88.3–100)
Conjunctival swab	11	0	0	11	NDET	NDET	29.4	100% (71.5–100)
**H5-negative clinical samples**								
Influenza A (H3N2) virus	21	0	21	21	NDET	28.7	30.9	100% (83.9–100)
Influenza A (H1N1) virus	17	0	17	17	NDET	28.8	30.2	100% (80.5–100)
Influenza B virus	3	0	0	3	NDET	NDET	29.4	100% (29.2–100)
Respiratory syncytial virus	7	0	0	7	NDET	NDET	30.1	100% (59.0–100)
SARS-CoV-2 virus	10	0	0	10	NDET	NDET	31.1	100% (69.2–100)

^*a*^ Specificity was assessed using influenza virus-negative specimens and residual specimens containing common respiratory pathogens. Samples without a positive result were labelled not detected (“NDET”).

Residual specimens known to contain other common respiratory pathogens were tested to assess specificity (Table 7). Importantly, we included non-H5 influenza A virus subtypes H3N2 (*n* = 21) and H1N1 (*n* = 17) identified by whole-genome sequencing (Table S11). We also included specimens containing influenza B virus (*n* = 3), respiratory syncytial virus (*n* = 7), and SARS-CoV-2 (*n* = 10). None of the specimens produced a positive result for the H5 target, demonstrating 100% negative agreement with prior clinical testing. The influenza A samples appropriately amplified the IAV M target, while samples containing other pathogens did not.

### Accuracy and precision

Combining all true positive and true negative results, the accuracy of the H5 target was 99.7% (95% CI: 98.6–100%) and the accuracy of the M target was 97.7% (95% CI: 95.6–99.0%) (Table 8).

**TABLE 8:** Accuracy of influenza virus targets^*a*^

		H5-negative clinical samples		H5-positive animal samples		Contrived H5-positive specimens	
		Test positive	Test negative	Test positive	Test negative	Test positive	Test negative
H5 target	Known positive	0	0	7	0	228	1
	Known negative	0	118	0	0	0	0
IAV M target	Known positive	38	0	6	1	222	7
	Known negative	0	80	0	0	0	0

^*a*^ Samples with known influenza status and known H5 status were used for validation. Due to the lack of H5-positive clinical samples available for validation, a combination of animal-source RNA samples and contrived specimens was used for H5-positive samples.

The precision of the multiplex RT-qPCR reaction was evaluated across 6 logs of input using a pool of IVT H5 RNA and NIST M RNA (Table S12). Templates were combined and diluted in AE buffer in a 10-fold series with four replicates per concentration. For the H5 target, the maximum coefficient of variation was 3.3% at a mean Ct value of 35.4 (estimated 58.0 copies per reaction). For the M target, the maximum coefficient of variation was 3.6% at a mean Ct value of 36.4 (estimated 13.0 copies per reaction).

Day-to-day and operator-to-operator variation were assessed by including a positive control reaction on each PCR plate (Table S13). Thirteen independent PCR plates run by 3 different operators over 5 days were analyzed. Mean Ct values were 30.4 for the H5 target (estimated 984 copies per reaction) and 28.3 for the M target (estimated 4,920 copies per reaction). Across all experiments, the total coefficient of variation was 2.1% for the H5 target and 2.4% for the M target. The day-to-day variation was 1.8% for the H5 target and 2.3% for the M target. The operator-to-operator variation was 1.1% for the H5 target and 1.2% for the M target.

### Comparison to CDC Influenza A/H5 Subtyping Kit

The assay’s performance was benchmarked against the CDC’s Influenza A/H5 Subtyping Kit to determine its suitability for surveillance and clinical applications. A comparison was made using the complete protocol of each assay, including extraction. In parallel, contrived positive specimens used to measure the initial LOD were extracted following each assay’s protocol. The CDC assay consistently produced higher Ct values than the multiplex assay (Fig. 1). The multiplex assay detected low-concentration samples more frequently than the singleplex CDC assay for both the H5 and pan-influenza A targets. The initial LOD of the CDC assay was estimated between 209 and 2,090 TCID50/mL for the H5a target, between 2,090 and 20,900 TCID50/mL for the H5b target, and between 209 and 2,090 TCID50/mL for the InfA target (Table S14). On confirmatory analysis using the more precise 4-fold dilution series, the LOD95 was estimated higher, between 3,120 and 12,500 TCID50/mL for the H5a target, between 780 and 3,120 TCID50/mL for the H5b target, and between 3,120 and 12,500 TCID50/mL for the InfA target (Table S15).

**Fig 3:**
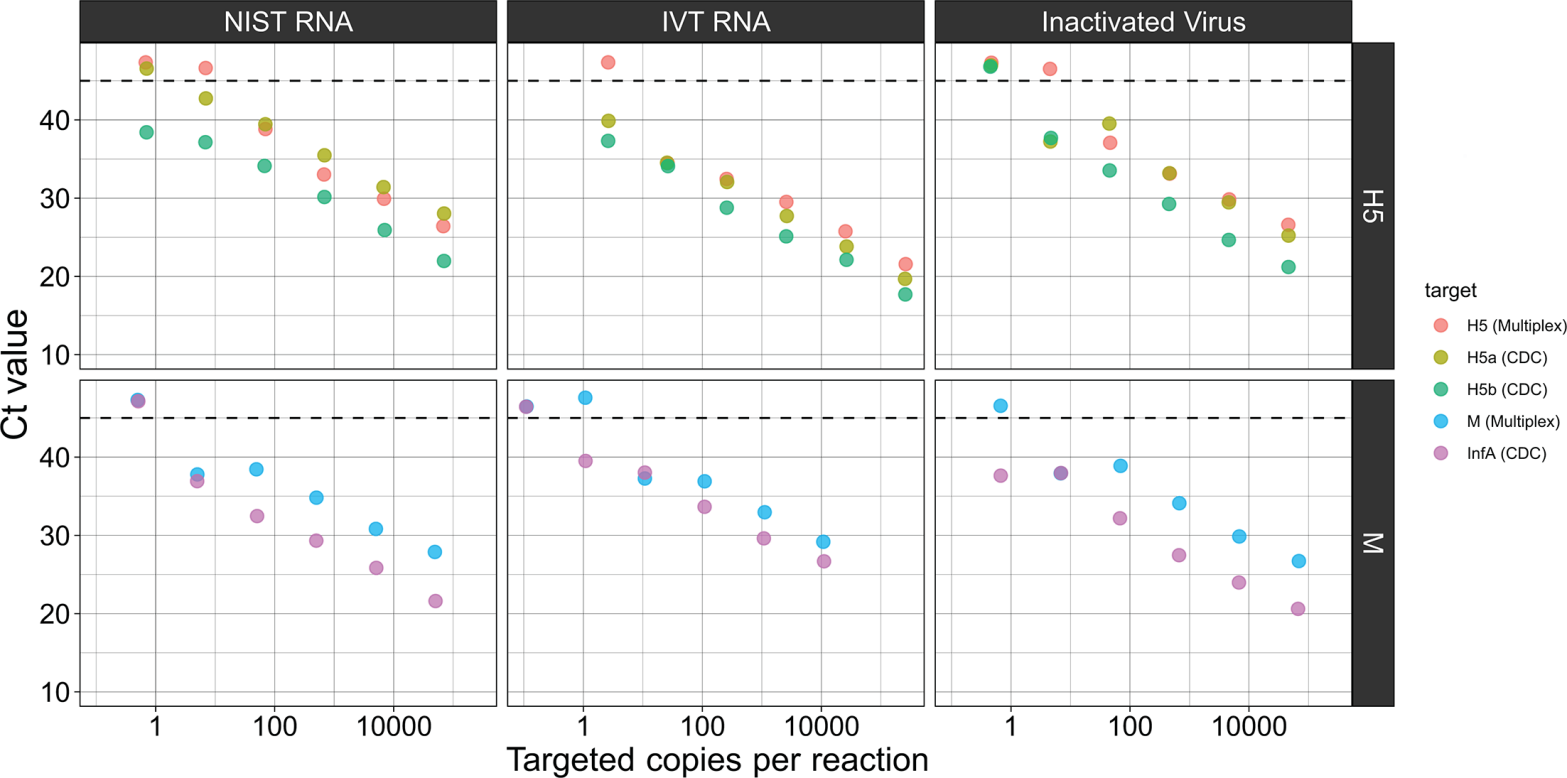
Comparison of multiplex RT-qPCR reaction and CDC RT-qPCR reactions. (*N* = 2). Ten-fold dilutions of purified RNA were tested in each assay’s RT-qPCR protocol. Targeted absolute copy number based on RT-ddPCR measurements is shown on the *X*-axis. Points appearing above the dashed line were not detected after 45 cycles of PCR. Reported values are the mean Ct value from two RT-qPCR technical replicates.

A comparison was also made of the RT-qPCR reactions on identical RNA templates. Ten-fold serial dilutions of the IVT RNA, NIST RNA, and inactivated virus templates were prepared, and the same aliquots were used for testing in parallel (Fig. 3). In this case, the CDC H5 Subtyping kit produced lower Ct values than the multiplex assay when tested on the same extracted RNA templates, especially for the pan-influenza A target, where Ct values differed by approximately 5 units. At the lower extreme of template concentration, the CDC H5 Subtyping Kit detected several samples that were not detected by the multiplex assay, suggesting higher sensitivity of the CDC RT-qPCR reaction. Above concentrations of 10 copies per reaction, corresponding to 500 copies/mL, both assays detected all samples tested.

### Retrospective subtyping of influenza A virus-positive samples collected March 2024 to February 2025

We retrospectively tested 590 residual samples that had been collected for influenza virus genomic surveillance between 11 March 2024 and 18 February 2025. These specimens tested positive for influenza A virus with at least one Ct value less than 31 on Cepheid Xpert Xpress SARS-CoV-2 Flu RSV plus or Hologic Panther Fusion Flu A/B/RSV assays at UW Medicine labs. 72.7% of specimens had Ct values below this threshold. The mean Ct value of all samples with a positive influenza A result on the Hologic assay across this study period (*n* = 348) was 27.6 with a standard deviation of 5.9 (Fig. S3A). On the dual-target Cepheid assay (*n* = 591), the mean influenza A Ct values were 24.7 for target Flu A1 (SD = 6.4) and 26.7 with 27 false-negative results for target Flu A2 (SD = 5.8) (Fig. S3B). Influenza virus whole-genome sequencing data were available for 171 specimens tested for retrospective H5 subtyping, and all contained seasonal influenza A virus subtypes H3N2 (*n* = 83) or H1N1 (*n* = 88) (Table S16).

**Fig 4:**
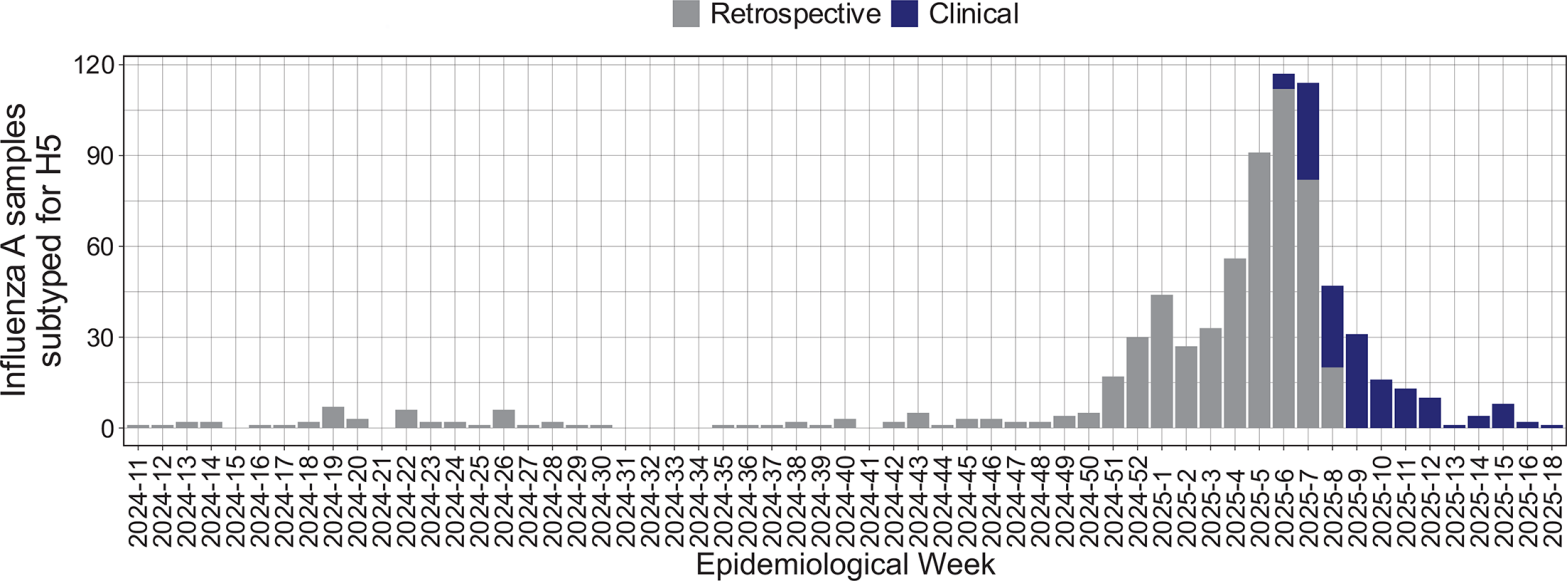
Influenza A virus-positive samples subtyped per epidemiological week. Gray bars indicate samples that were collected for influenza virus surveillance and retrospectively subtyped. Blue bars indicate samples that were ordered for clinical testing after implementation at UW hospitals.

At least one sample was available for 43 out of 50 weeks across the study period for retrospective H5 subtyping (Fig. 4). The median number of samples subtyped per week was 2 (IQR: 1–6). During the winter influenza season (epidemiological weeks 2024-51 to 2025-08), a median of 44 samples per week were subtyped with 512 samples in total. None of the samples tested positive for the H5 target (Table 9). Almost all samples (98.8%) tested positive for the pan-influenza A virus M target.

**TABLE 9:** H5 testing of influenza A virus-positive samples^*a*^

Specimen type	*N*	H5 positive	IAV M positive	Internal control positive
Retrospective subtyping	590	0	583	590
Routine clinical care	150	0	126	150

^*a*^ Retrospective subtyping refers to residual specimens tested for surveillance purposes. Routine clinical care refers to H5 subtyping tests ordered prospectively once the validated clinical assay was implemented.

The patient population from whom these samples were collected (Table 7) consisted predominantly of adults, with a median age of 40 years (IQR: 26–59). Most samples were collected in emergency departments (*n* = 379) or outpatient clinics (*n* = 150). Eighteen samples were sourced from outside of the UW Medicine health system. Based on the address provided at the time of clinical testing, approximately 92% of all samples were obtained from patients residing in Seattle and Western Washington (Table S17) based on the first three digits of patients’ home zip code. Of 590 patients, 388 came from Seattle’s urban core. An additional 156 patients came from the greater Seattle metropolitan area or Western Washington. Beyond Western Washington, 12 samples came from patients residing in other parts of Washington State or Alaska. The remaining 26 samples originated from patients with home addresses across the United States.

### Prospective clinical subtyping of influenza A virus-positive samples (February 2025 to April 2025)

The H5 assay was implemented at UW Medicine on 6 February 2025. One hundred fifty H5 subtyping tests were ordered on influenza A virus-positive patients during routine clinical care from 6 February 2025 to 30 April 2025. None of these samples tested positive for H5 influenza virus. The majority (84.0%) tested positive for the pan-influenza A virus target M. Although the clinical tests for influenza A available through UW Medicine (Cepheid Xpert Xpress SARS-CoV-2 Flu RSV plus and Hologic Panther Fusion Flu A/B/RSV assays) are reported qualitatively, Ct values were available internally for 105 patients, including 16 that had false-negative M target results on subsequent subtyping (Fig. S4). Of these, 4 samples also had false-negative Flu A2 targets on the Cepheid assay. False-negative M target results were almost exclusively found near the lower end of the viral load range, with mean initial Ct values of 34.7 and standard deviation of 5.2 for the Hologic or Cepheid influenza A targets.

Across this 12-week period, the median number of H5 subtyping test orders was 9 per week (IQR: 3.5–18.8) and the maximum was 32 per week. Most test orders (*n* = 104) came from emergency departments or were ordered for patients admitted to an inpatient service (*n* = 45); one order was made from an outpatient clinic. The median age of patients who received an H5 subtyping test as part of clinical care was 62 years old (IQR: 46.5–75), which is significantly older than patients in the retrospective subtyping cohort (*P* < 0.01 by Wilcoxon rank-sum test) (Table 10). There were also more samples collected from male patients in this cohort than in the retrospective subtyping cohort (52.0% vs 50.3%), although this difference was not statistically significant (*P* = 0.75, chi-squared test).

**TABLE 10:** Demographic information related to samples tested retrospectively for H5 influenza virus^*a*^

	Retrospective subtyping		Routine clinical care	
	*N*	%	*N*	%
**All samples**	**590**	** **	**150**	** **
**Age**				
0–4 years	28	4.7	1	0.7
5–17 years	38	6.4	2	1.3
18–49 years	305	51.7	40	26.7
50–64 years	103	17.5	39	26.0
≥65 years	116	19.7	68	45.3
**Race**				
American Indian or Alaska Native	13	2.2	5	3.3
Asian	57	9.7	8	5.3
Black or African American	94	15.9	20	13.3
Native Hawaiian or Pacific Islander	7	1.2	4	2.7
White	334	56.6	107	71.3
Another race	23	3.9	2	1.3
Not available	62	10.5	4	2.7
**Ethnicity**				
Hispanic or Latino	76	12.9	17	11.3
Not Hispanic or Latino	455	77.1	128	85.3
Not available	59	10.0	5	3.3
**Sex**				
Female	291	49.3	71	47.3
Male	297	50.3	78	52.0
Not available	2	0.3	1	0.7
**Source**				
Emergency department	379	64.2	104	69.3
Inpatient	43	7.3	45	30.0
Outpatient	150	25.4	1	0.7
Outside source (not from UW Medicine)	18	3.1	0	0.0

^*a*^ Demographic information was obtained from the electronic health record. “Not Available” includes data values “N/A,” “Declined to answer,” and “Unknown”.

## DISCUSSION

Here, we report the design, validation, and clinical implementation of an RT-qPCR assay to subtype H5 influenza A virus in nasopharyngeal, nasal, and conjunctival swab specimens. The assay is sensitive, with a LOD of 250 copies/mL, and specific, with no cross-reactivity to common respiratory pathogens, including other influenza A virus subtypes. Recently, several other groups have validated and implemented laboratory-developed tests for H5 subtyping, though little published performance characteristic data are available for comparison (19–21). The sensitivity of our assay is comparable to that of the assay recently published by Sahoo et al., with both assays reporting limits of detection below 5 copies per reaction (19).

It is difficult to compare these values to the LOD reported for the CDC’s H5 subtyping kit, ~250 EID50/mL, as this value is reported in units of egg infectious dose rather than absolute copy number. In both tissue culture models (31) and human infections (32), the number of template molecules detectable by RT-qPCR does not consistently correlate with viral titer because the ratio of non-infectious viral particles varies greatly between individual infections. However, when the same specimens were tested side-by-side, our multiplex assay demonstrated higher sensitivity than the CDC H5 Subtyping Kit. This difference was due to the efficiency of RNA extraction, as the CDC RT-qPCR reaction demonstrated higher sensitivity when identical isolated RNA templates were tested. In addition to high sensitivity, the multiplex format of our assay requires just 1 reaction per test, simplifying handling and reducing the cost of the test compared to the 4-well design of the CDC assay.

Given the preponderance of conjunctivitis reported in human cases related to the current outbreak, we validated the assay’s compatibility with conjunctival swab specimens. Although the CDC H5 subtyping kit is authorized for use with conjunctival swab specimens (33), to the best of our knowledge, no published validation studies have included this specimen type. We also validated the assay’s ability to detect both circulating genotypes that have caused human cases, B3.13 and D1.1.

Following validation, we tested 740 specimens from UW Medicine collected between March 2024 and April 2025 and identified no infections with subtype H5 virus. Importantly, we used the test to distinguish between H5 influenza virus and seasonal influenza viruses during the 2024–2025 influenza virus season, and influenza virus sequencing data were available for 157 of these specimens. This is the first surveillance study performed during a period of high seasonal influenza virus activity (33) since the outbreak began and complements a recent study by Adams et al. that detected no H5N1 infections during a period of low seasonal influenza virus activity (34). Our data set includes both retrospective testing of residual specimens (*n* = 590) and clinical testing ordered during routine medical care after the assay was implemented at UW Medicine (*n* = 150). This surveillance was performed in Washington State, which to date has experienced the second-highest number of human cases of H5N1 influenza infection in the United States.

Our results come with several limitations. First, the assay is designed to maximize the sensitivity of the H5 targets at the expense of the M target. Because of this, the M target is less sensitive, and we have observed false-negative results on samples that are known to be influenza A virus-positive. This limitation is ameliorated by the ability of all influenza tests to detect H5N1 as influenza virus, and the indication of the subtyping assay to be used with known influenza virus-positive specimens. Second, the probe “H5 Probe 1” carries mismatches to the majority of recent H5 sequences from North America. This occurred because initially, material for validation was extremely limited, and probes were designed to bind the only inactivated H5N1 reference material available (BEI NR-59421), which encoded an *HA* sequence from 2009. Since that time, updated inactivated H5N1 reference material (BEI NR-59886) has become available and was used for validation, highlighting the importance of making inactivated viral material available that can be used for BSL-2 validation activities. While our probe functions with contemporary H5N1 genotypes, future evolution might diminish probe binding and necessitate a revised probe sequence. Third, while the exogenous internal control we add during extraction controls for RT-qPCR reaction performance, it does not control for specimen adequacy (e.g., confirm that a specimen was loaded into extraction), and an endogenous control target would be useful for this purpose in the future. Finally, although our surveillance data represent the largest set of influenza A virus-positive specimens tested for H5 in the literature since the outbreak began, almost all tested specimens originated from Seattle, Washington. Sampling from more rural regions, where patients are more likely to have exposure to livestock or avian species, is critical for both rapid detection of zoonotic infections and assessing point prevalence in a potentially higher-risk region. With engagement and collaboration with clinicians and public health departments in rural areas, the clinically reportable H5 subtyping assay described here can help in these efforts.

Our data are most useful for understanding the prevalence of H5 infections during the 2024–2025 annual influenza virus season in a single academic medical system. We detected no cases of subtype H5 infection within the population of patients infected with influenza A viruses. As such, we did not detect evidence of any evolutionary events that can occur in the presence of co-circulation, such as recombination between H5 viruses and seasonal influenza A viruses (1). Our conclusions are in agreement with data from both the national influenza surveillance program, which has identified very few cases through non-specific respiratory disease surveillance (7), and the recent Adams et al. study, which found no H5 cases by screening clinical respiratory samples collected outside of the annual influenza season (34). Taken together, these data support current public health guidance indicating that H5 infection risk remains almost entirely associated with exposure to infected animals. Focused testing of patients with epidemiological risk factors, such as farm workers, and prompt treatment with antiviral medications remain cornerstones of clinical management (35). However, as H5N1 influenza virus is expected to spread in animal species in the United States for the foreseeable future, integration of routine H5 testing into health systems remains critical to monitor for future changes to the epidemiology of this outbreak.

## Data Availability

Viral sequences referenced in this manuscript are available under BioProject PRJNA1232193.

## SUPPLEMENTAL MATERIAL

Figure S1 jcm.00415-25-s0001.pdf H5 Primer and probe binding sites.

Figure S2 jcm.00415-25-s0002.pdf Relationship between RT-qPCR Ct value and RT-ddPCR absolute copy number.

Figure S3 jcm.00415-25-s0003.pdf Ct values from influenza A-positive clinical tests from March 2024 to February 2025.

Figure S4 jcm.00415-25-s0004.pdf Comparison of pan-influenza A target Ct values from clinically ordered tests.

Supplementary File 1 jcm.00415-25-s0005.txt H5 gBlock construct sequence used in validation.

Supplemental tables jcm.00415-25-s0006.xlsx Tables S1 to S17.

ASM does not own the copyrights to Supplemental Material that may be linked to, or accessed through, an article. The authors have granted ASM a non-exclusive, world-wide license to publish the Supplemental Material files. Please contact the corresponding author directly for reuse.
