# Supplementary material for: Validation of H5 influenza virus subtyping RT-qPCR assay and low prevalence of H5 detection in 2024–2025 influenza virus season
Source: J Clin Microbiol. 2025 Oct 21;63(11):e00415-25. doi: 10.1128/jcm.00415-25 (PMC12607698; doi:10.1128/jcm.00415-25)
Supplement: Figure S1 — H5 Primer and probe binding sites. [file jcm.00415-25-s0001.pdf]

	**H5 Forward 1**	**H5 Probe 1**	**H5 Reverse 1**
	TGGAAAGTGTGAGAAATGGGACGT	TGACTACCCGCAGTATTCAGAAGAAGCAAGACTAA	CAGCGGCAAGTTCCCTAGCA
**NIST RNA**	....	.........T.....................T...	....
**IVT RNA**	....	.........T.....................T...	.......G............
**BEI Inactivated Virus**	....	.........T.....................T...	....
**CDC Inactivated Virus**	....	.........T....................GT...	.......G............
**Genotype B3.13**	....	.........T.....................T...	....
**Genotype D1.1**	....	.........T....................GT...	.......G............

	**H5 Forward 2**	**H5 Probe 2**	**H5 Reverse 2**
	TGGGTACCATCATAGCAATGAGCA	TGGGTACGCTGCGGACAAAGAATCCA	TTTGAGGCAGTTGGAAGGGAGTT
**NIST RNA**	....	....	....
**IVT RNA**	....	............A.............	....
**BEI Inactivated Virus**	....	....	....
**CDC Inactivated Virus**	....	............A.............	....
**Genotype B3.13**	....	....	....
**Genotype D1.1**	....	............A.............	....

Figure S1: H5 Primer and probe binding sites. NIST RNA (GenBank: N90JTPPO), IVT RNA (GenBank: LC730539), BEI Inactivated Virus (GenBank: PP836471) and CDC Inactivated Virus (GenBank: PQ573554) refer to *HA* templates utilized in the validation studies. B3.13 HA (GenBank: PP692142) and D1.1 HA (GenBank: PQ832645) are representative sequences of each genotype.
