# Supplementary material for: Validation of H5 influenza virus subtyping RT-qPCR assay and low prevalence of H5 detection in 2024–2025 influenza virus season
Source: J Clin Microbiol. 2025 Oct 21;63(11):e00415-25. doi: 10.1128/jcm.00415-25 (PMC12607698; doi:10.1128/jcm.00415-25)
Supplement: Figure S2 — Relationship between RT-qPCR Ct value and RT-ddPCR absolute copy number. [file jcm.00415-25-s0002.pdf]

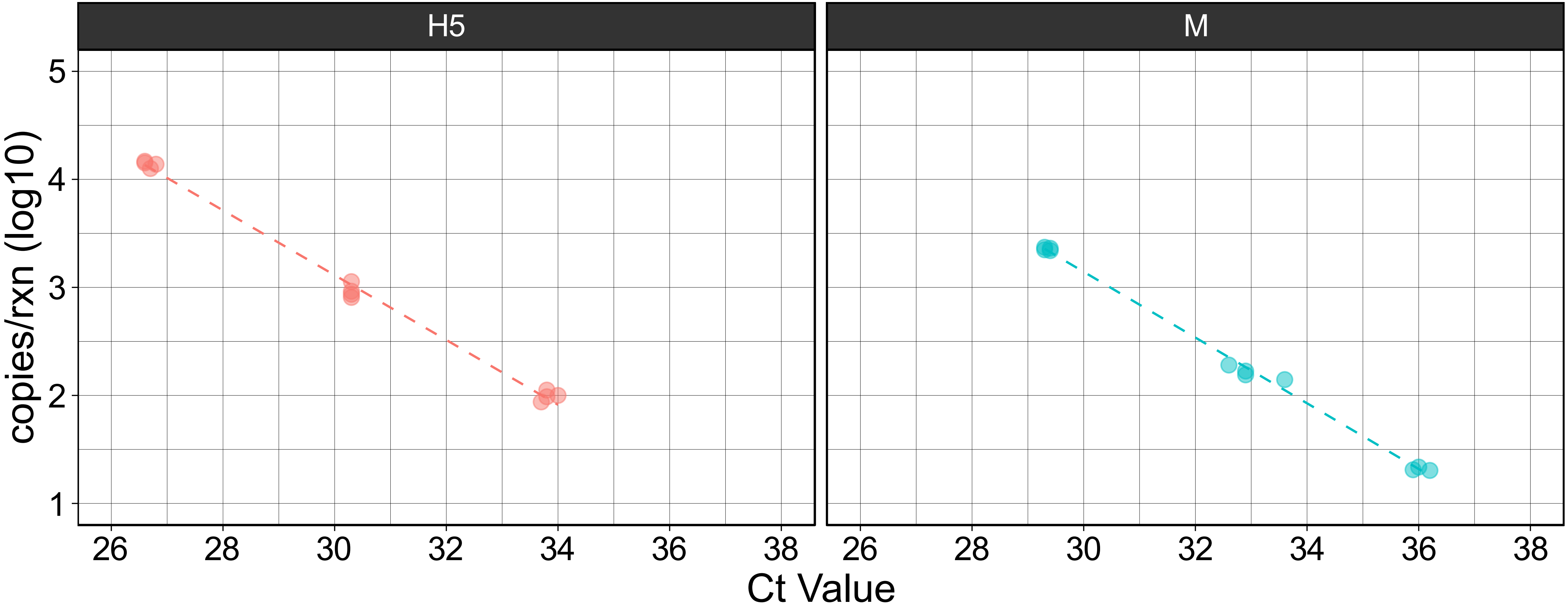

Figure S2: Relationship between Ct value and absolute copy number. N=4. RNA extracted from H5N1 virus A/Washington/239/2024 was serially diluted and tested simultaneously by RT-qPCR and ddPCR.
