# Supplementary material for: Validation of H5 influenza virus subtyping RT-qPCR assay and low prevalence of H5 detection in 2024–2025 influenza virus season
Source: J Clin Microbiol. 2025 Oct 21;63(11):e00415-25. doi: 10.1128/jcm.00415-25 (PMC12607698; doi:10.1128/jcm.00415-25)
Supplement: Figure S3 — Ct values from influenza A-positive clinical tests from March 2024 to February 2025. [file jcm.00415-25-s0003.pdf]

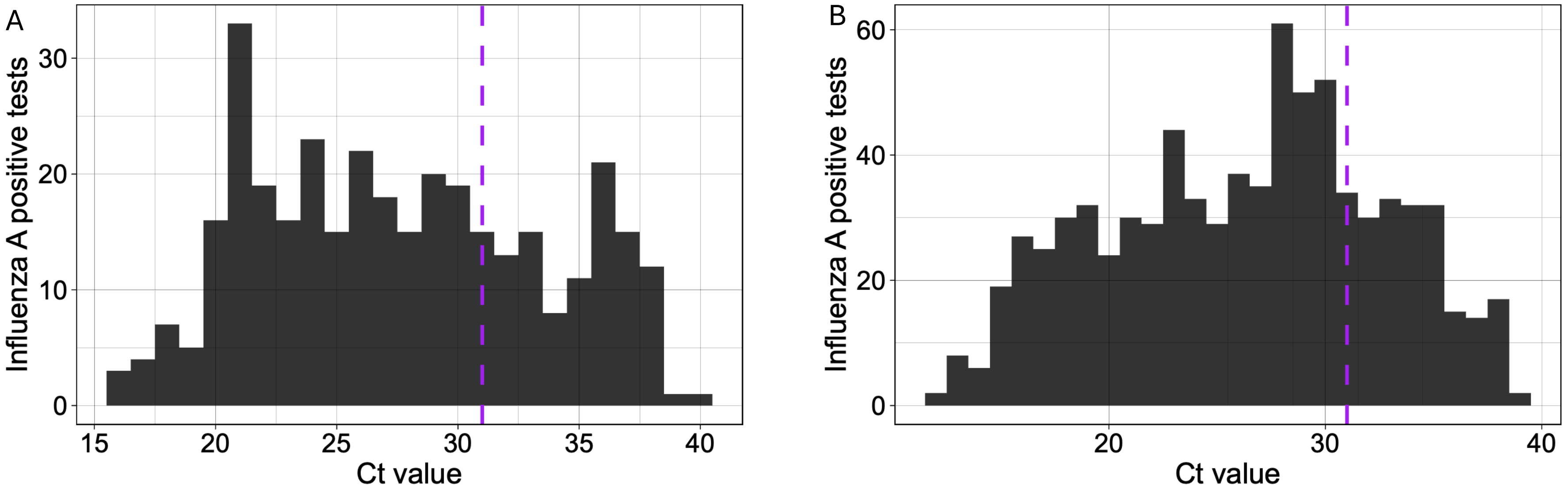

Figure S3: Ct values of influenza-positive clinical results collected between March 2024 and February 2025. A) Hologic Panther Fusion Flu A/B/RSV assay (n=348).B) Cepheid Xpert® Xpress SARS-CoV-2 Flu RSV plus (Flu A1 target) (n=591). Dashed line at Ct value 31, which is the threshold for sample collection for genomic surveillance. Residual genomic surveillance samples were used for retrospective subtyping in this study.
