# Supplementary material for: Validation of H5 influenza virus subtyping RT-qPCR assay and low prevalence of H5 detection in 2024–2025 influenza virus season
Source: J Clin Microbiol. 2025 Oct 21;63(11):e00415-25. doi: 10.1128/jcm.00415-25 (PMC12607698; doi:10.1128/jcm.00415-25)
Supplement: Figure S4 — Comparison of pan-influenza A target Ct values from clinically ordered tests. [file jcm.00415-25-s0004.pdf]

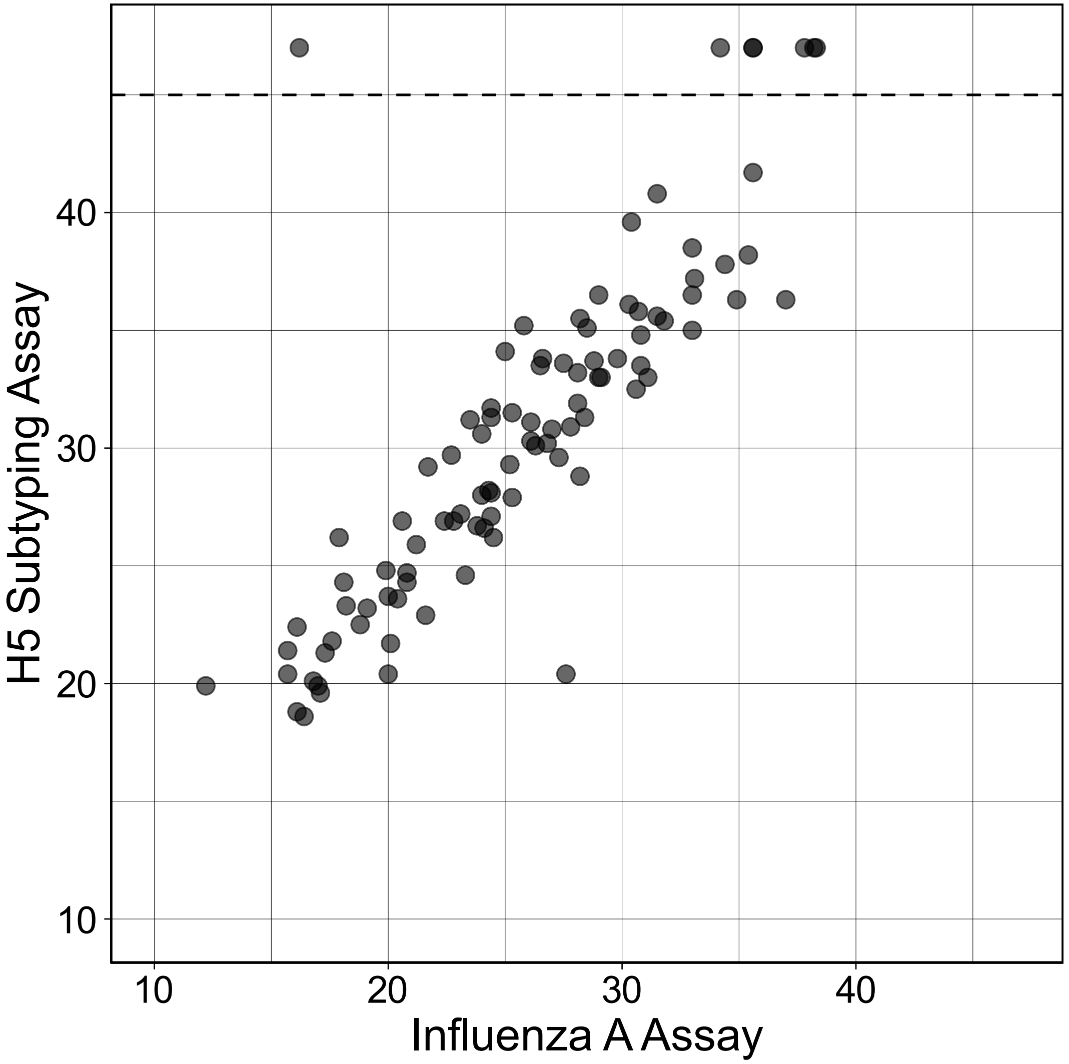

Figure S4: Comparison of pan-influenza A target Ct values from clinically ordered tests. N=105. Patients with both clinically ordered H5 subtyping assay results (M target) and prior influenza clinical testing Ct values available. Initial influenza testing was performed with either the Cepheid Xpert® Xpress SARS-CoV-2 Flu RSV plus (Flu A1 target) or Hologic Panther Fusion Flu A/B/RSV (Flu A target). Points appearing above the dashed line were not detected after 45 cycles of PCR.
